# Quality of life among multiple sclerosis patients in Saudi Arabia

**DOI:** 10.17712/nsj.2017.4.20170273

**Published:** 2017-10

**Authors:** Hussein A. Algahtani, Bader H. Shirah, Faris A. Alzahrani, Hind A. Abobaker, Nebras A. Alghanaim, Juan S. Manlangit

**Affiliations:** *From the Neurology Section (Algahtani), Department of Medicine, King Abdulaziz Medical City, from King Abdullah International Medical Research Center (Shirah), King Saud bin Abdulaziz University for Health Sciences, and from King Saud bin Abdulaziz University for Health Sciences (Algahtani, Alzahrani, Abobaker, Alghanaim, Manlangit Jr), Jeddah, Kingdom of Saudi Arabia*

## Abstract

**Objective:**

To assess quality of life in multiple sclerosis (MS) patients and determine the factors associated with levels of quality of life in MS patients in a public hospital in Saudi Arabia.

**Methods:**

A cross-sectional study was conducted from June 2016 to April 2017 in King Abdulaziz Medical City, Jeddah, Kingdom of Saudi Arabia. Multiple sclerosis patients attending the outpatient and inpatient services were approached and recruited to participate in the study. The Arabic version of EuroQOL-5 Dimensions instrument (EQ-5D) was utilized for the assessment of MS patients quality of life.

**Results:**

Data on quality of life were obtained from 292 patients. The reported quality of life of MS patients as measured by the EQ-5D index value score was 0.31±0.51 and the EQ-VAS score was 73.87±23.41, respectively. It was found that quality of life determined numerically in the EQ-5D index value and EQ-VAS deteriorates proportionally according to the disease duration.

**Conclusion:**

Multiple sclerosis is associated with a considerable effect on the patients quality of life. It continues to be challenging to manage both medically and psychosocially. Clinicians should consider the assessment of quality of life as routine practice along with the other important measures including symptomatic evaluation, laboratory tests, and neuroimaging to provide a holistic care of their MS patients.

Multiple sclerosis (MS) is a chronic demyelinating disease of the central nervous system (CNS) characterized by loss of sensory and motor functions as a result of inflammation, demyelination, and axonal damage.^1^ Although MS is one of the most common causes of disability in young adults, its etiology remains unknown. It is thought to be an immune-mediated disorder that arises from a combination of genetic susceptibility and environmental factors acting from gestation to early adulthood.^2^ The prevalence of MS is variable globally ranging from 15/100,000 to 250/100,000 with approximately 2.5 million total cases affected worldwide.^3^ Clinically, MS patients experience recurrent episodes of neurological impairment (relapses), and the disease becomes chronic and progressive with time, which subsequently leads to motor disability and cognitive deficits.^4^ Assessment of quality of life is one of the most important methods to measure the impact of the disease on the patient. It also helps to monitor the clinical status of the patients and to measure the treatment efficacy.^5^ In this study, we aim to assess quality of life in MS patients and determine the factors associated with levels of quality of life in MS patients in a public hospital in Saudi Arabia. The results obtained will help to improve healthcare for MS patients and assess the social impact and economic cost of this disease by indicating their specific needs for pharmacotherapy, rehabilitation, and multidisciplinary care.

## Methods

This is a cross-sectional study carried out in King Abdulaziz Medical City, Jeddah, Kingdom of Saudi Arabia. Data collection started from June 2016 to April 2017. Patients who have MS and attending the inpatient and outpatient services were asked to participate in the study. From a total of 352 MS patients, 292 (82.95%) participated in the study. Face to face interview was conducted by the neurology consultants to assure the credibility of the interview process and correctness of the collected data. Collected data from all MS patients includes their demographic profile (i.e. age and gender), illness information (i.e. form of MS, age at disease onset, duration of illness, and current treatment), and health status by utilizing the EuroQOL-5 Dimensions (EQ-5D) questionnaire in Arabic version.

The validity and reliability of the Arabic version of the EQ-5D questionnaire have been demonstrated for the general Arabian population.^6^ The EQ-5D is a generic instrument that has been widely used for generalizability and comparability on patients’ general well-being and ability to function in everyday life. This is comprised of 2 parts; the first part is the EQ-5D descriptive system that involved patient self-reporting of their health status in terms of 5 dimensions: mobility, self-care, usual activates, pain/discomfort, and anxiety/depression. Each dimension has 5 levels: 1=no problems, 2=slight problems, 3=moderate problems, 4=severe problems and 5=extreme problems. Since the numerals 1-5 have no arithmetic properties and cannot be used as a cardinal score,^7^ the EQ-5D index value for MS patients quality of life was calculated based on United Kingdom (UK) value set in Crosswalk Index Value Calculator.^8^ The second part is the EuroQol Visual Analog Scale (EQ-VAS), which is a 20 cm visual vertical scale labeled “the best health you can imagine=100” and “the worst health you can imagine=0” to rate themselves about their current health status. The EQ-VAS, however, does not influence the EQ-5D-5L index but provides an additional information about their quality of life.^7^ This study was registered with EuroQoL, and permission was given for its use (ID: 7305, approval date: 5 December 2014).

Descriptive statistics such as frequency, percentage, mean and standard deviation (SD) were used to describe and synthesize MS patients’ demographic data, illness information, and reported problems for each level for each dimension of EQ-5D. Inferential statistics were then applied to determine differences in MS patients’ EQ-5D index value when grouped according to demographic variables (i.e. gender) and illness information (i.e. duration of illness). Independent t-test was utilized to test the significance of differences between nominal variables (i.e. gender) and one-way ANOVA to test significant differences between more than 2 groups in interval data (i.e. duration of illness). The Pearson correlation coefficient was used to assess the correlation between the reported EQ-VAS scores and EQ-5D-5L index values. Multiple linear regression was carried out to identify which variables were significantly associated with the quality of life of MS patients (dependent variable). The independent variables were age, sex, duration of illness, and on treatment or not on treatment. The Statistical Package for Social Sciences (SPSS) version 21.0 was used for data analysis. The significance level was predetermined at *p*-level<0.05 for all tests.

All questionnaires were anonymous, and confidentiality of information was ensured. Instructions to complete the questionnaire and confidentiality issues were explained on the cover page of each questionnaire. Completion of the questionnaire was considered as a consent to participate in the study. This study was approved by the Institutional Review Board (IRB) of King Abdullah International Medical Research Center (KAIMRC).

## Results

Data on quality of life were obtained from 292 patients. Total mean age for the entire group of patients was 35.95±10.26 years (range 14-70 years), with a mean age of the women being 35.96±10.59 years and mean age of the men 35.94±9.53 years. Females represented 69.2% of the study population (202 patients), with males represented 30.8% (90 patients), with a gender ratio of 2.24:1. The mean age at onset of the disease was 28.43±9.42 years old; range 11-63 years. The mean duration of the disease was 7.63±6.17 years; ranging from 2 weeks to 35 years. Detailed patients’ demographic data are presented in **Table 1**.

**Table 1:** Baseline demographics of multiple sclerosis patients.

Demographics	Women	Men	All Patients
Patients n (%)	202 (69.2)	90 (30.8)	292
Women vs Men	-	-	2.24:1 ratio
Age (years)	35.96 ±10.59	35.94 ±9.53	35.95±10.26)
Age at disease onset	28.33 ±9.69	28.66 8.83±	28.43±9.42
Duration of illness (years)	7.68±6.31	7.52 ±5.88	7.63±6.17
***Form of multiple sclerosis n (%)***			
RRMS	78 (26.7)	181 (62)	259 (88.7)
SPMS	6 (2.05)	6 (2.05)	12 (4.1)
PPMS	6 (2.05)	15 (5.1)	21(7.2)
EQ-5D index	0.27 (0.51)	0.40 (0.50)	0.31 (0.51)
EQ-VAS	73.24 (22.52)	75.28 (25.38)	73.87(23.41)

SD - standard deviation PRMS - Progressive-relapsing MS, SPMS - secondary progressive MS, PPMS - primary progressive MS, EQ-5D - EuroQOL-5 Dimensions instrument

Most of our patients included in the study 259 (88.7%) had a relapsing-remitting form of the disease, 12 (4.1%) were secondary progressive, and 21 (7.2%) were primary progressive. Most of the patients 244 (83.6%) were receiving disease modifying drugs and 48 (16.4%) were not on treatment.

**Figure 1:**
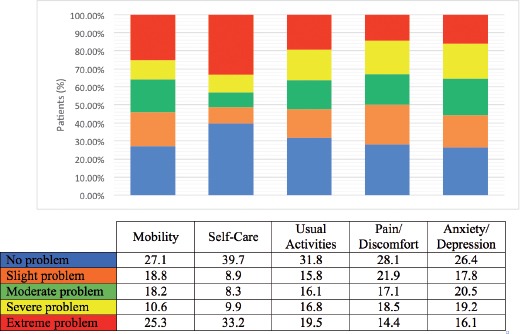
Subjective assessment of EQ-5D dimensions instrument.

**Figure 2:**
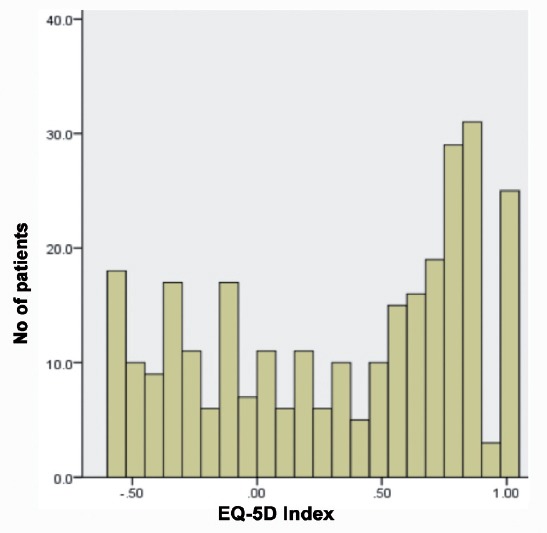
Frequency Distribution Histogram of EuroQOL-5 dimensions instrument index. EQ-5D- EuroQOL-5 dimensions

The results of the subjective assessment of the EQ-5D are shown in **Figure 1**. The EQ-5D index was 0.27±0.51 for the females, while it was 0.40±0.50 for the males. The EQ-5D index for all patients was 0.31±0.51. No significant difference was found between genders (*p*=0.6). It was found that quality of life determined numerically in the EQ-5D deteriorates proportionally according to the duration of the disease (**Figure 2**).

**Figure 3:**
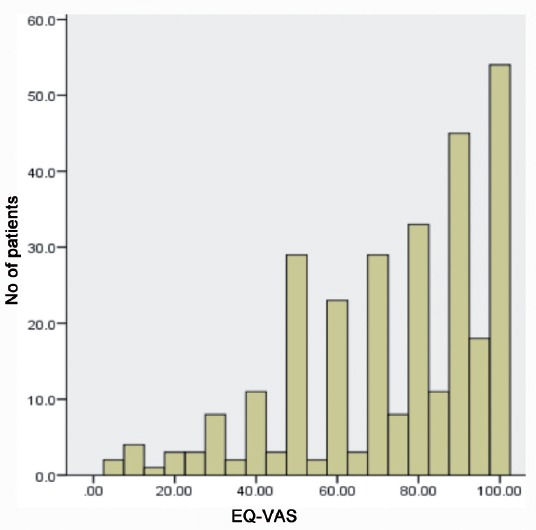
Frequency Distribution Histogram of EuroQol Visual Analog Scale. EQ-VAS - EuroQol Visual Analog Scale

**Figure 4:**
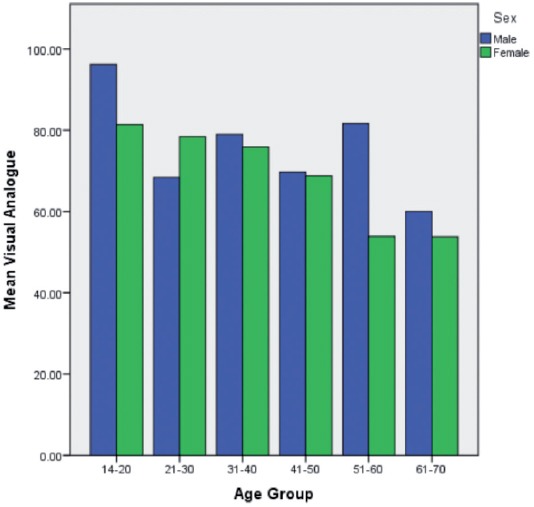
Multiple sclerosis patients EQ-VAS mean scores by age group and gender.

**Figure 5:**
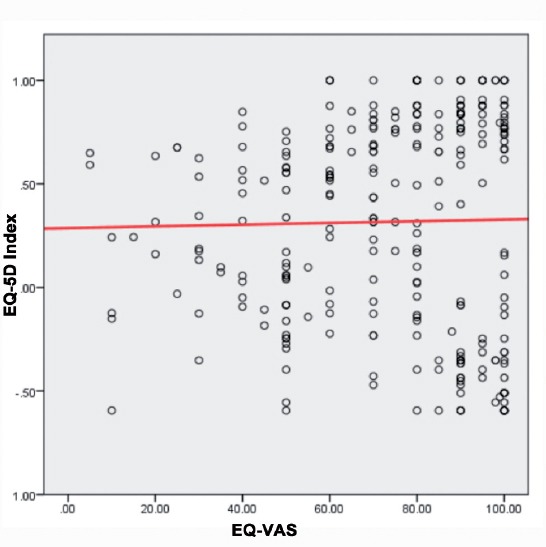
Correlation between EQ-5D index and EQ-VAS. EQ-5D- EuroQOL-5 Dimensions instrument

The reported quality of life of MS patients as measured by the EQ-5D index value was 0.31±0.51 and the EQ-VAS score was 73.87±23.41. The distribution of reported no problems across dimensions of quality of life was as follows: mobility 79 (27.1%), usual activities 93 (31.8%), self-care 116 (39.7%), pain/discomfort 82 (28.1%), and anxiety/depression 77 (26.4%) (**Table 2**). A total of 178 states of health were reported by the participants. We found that 25 (8.6%) patients reported no problems for any dimension and 14 (4.8%) patients reported extreme problems in all 5 dimensions. The mean score of the EQ-VAS for the females was 73.24±22.52, while it was 75.28±25.38 for the males. No significant difference was found between genders (*p*=0.21) (**Figure 4**). The histogram distribution of the numerical result EQ-VAS is shown in **Figure 3**. The correlation between the EQ-5D index and EQ-VAS was not significant (r=0.019, *p*>.005) (**Figure 5**). However, we found out that age, sex, duration of illness, and treatment or on treatment have no significant association with the EQ-5D index value of quality of life among MS patients (**Table 3**).

**Table 2:** Frequency distribution of MS patients reporting levels 1 to 5 by dimensions and by age group.

EQ-5D dimensions	Age group						Total
	14-20	21-30	31-40	41-50	51-60	61-70	
***Mobility level***							
1	8	23	25	14	9	0	79
2	0	9	25	19	1	1	55
3	1	15	19	13	2	3	53
4	0	6	15	8	2	0	31
5	2	29	24	12	5	2	74
***Self-Care level***							
1	8	31	43	23	10	1	116
2	0	4	13	9	0	0	26
3	0	7	9	7	1	0	24
4	0	2	10	9	6	2	29
5	3	38	33	18	2	3	97
***Usual Activity level***							
1	6	25	38	14	9	1	93
2	0	11	16	19	0	0	46
3	1	9	23	10	3	1	47
4	3	17	10	13	4	2	49
5	1	20	21	10	3	2	57
***Pain/Discomfort level***							
1	5	19	35	17	6	0	82
2	3	17	24	12	6	2	64
3	0	11	16	18	2	3	50
4	1	20	14	14	5	0	54
5	2	15	19	5	0	1	42
***Anxiety/Depression level***							
1	6	18	28	21	3	1	77
2	1	12	23	11	4	1	52
3	1	18	21	13	5	2	60
4	3	21	12	14	5	1	56
5	0	13	24	7	2	1	47

MS - multiple sclerosis, EQ-5D- EuroQOL-5 Dimensions instrument

**Table 3:** Multiple linear regression analysis of association between demographic and illness information variables to EQ-5D index value score.

Independent Variables	Dependent variable (EQ-5D index score)		
	Unstandardized coefficients B	Standardized coefficients B	*P*-value
Age	.005	.090	.170
Gender	-.121	-.109	.064
Duration of Illness in years	-.009	-.105	.107
On treatment and not on treatment	.044	.032	.588

B - Beta, EQ-5D - EuroQOL-5 Dimensions instrument

## Discussion

In this study, we elected to utilize the EQ-5D questionnaire due to its very simple format. It takes a little time to be filled up and most of the participants find it easy to be filled. Although it is a very simple questionnaire, its design does not negatively affect its usefulness and reliability. There are other questionnaires that are available in Arabic such as the MusiQoL and the SF-36. The Middle East MS Advisory Group recommends routine assessments of quality of life using the MusiQoL and the SF-36 in addition to other supplementary information.^9^

Quality of life is a broad concept that is affected by multiple parameters other than the patient’s health status. These parameters include level of independence, social setting, and psychological state. The assessment of quality of life has been increasingly recognized as an important aspect of MS research.^10^ Several studies reported in the literature regarding quality of life in MS patients showed an obvious deterioration as compared to the healthy population. In addition, MS was reported to be associated with a worse quality of life as compared to patients with other chronic diseases such as diabetes, congestive heart failure, myocardial infarction, hypertension, or depression.^11^

Multiple sclerosis affects the patient in a variety of aspects apart from the physical illness. These aspects include the psychological state, pain, vitality, sexual dysfunction, fatigue, financial problems, employment difficulties, and the perception of MS within their community.^12^ Depression and loss of cognitive function usually affect the employment status and social functioning of the patient. These 2 symptoms were reported by patients with MS as important determinants of their quality of life, and some patients believe that these symptoms are more important than their physical impairments. Depression may be corresponding to the pathologic changes in the CNS, mainly in the left frontal and temporal lobes.^13^ In our study, more than 50% of the patients reported moderate to extreme problems with depression. Our results indicate that depression highly correlates with quality of life.

Pain in MS patients is a common symptom estimated to affect more than 40% of patients. The pathophysiology explaining the pain in MS patients remains incompletely understood, but it is thought to be related to demyelinating lesions involving certain neuroanatomic pathways such as the spinothalamic and quintothalamic pathways, abnormal impulses through motor axons, development of an acquired channelopathy in affected nerves, or inflammatory immune mechanisms of the glial cell.^14^ Painful conditions in MS patients include central and peripheral neuropathy, trigeminal neuralgia, glossopharyngeal neuralgia, migraines, complex regional pain syndrome, painful tonic spasms, and transverse myelitis. MS relapses also cause pain, and patients usually report paroxysmal dystonia and neuropathic pain during the relapses.^15^ Pain is associated a reduced quality of life in patients with MS, and about 12% report pain as their worst symptom.^16^ In our patients, about 50% of the patients reported moderate to extreme problems with pain, which is close to other studies.

The use of disease modifying drugs has been reported to affect the quality of life. The variability of the results may be due to the availability of a variety of medications with variable side effects. The frequent self-injection with its associated side effects including injection-site reactions and flu-like symptoms may negatively impact the quality of life. Another important point to be illustrated is that the benefits of these medications may not be obvious to patients which may lead to low adherence and further relapses that will also affect the quality of life negatively.^17^ In our study, no statistically significant difference was found between the patients who were on disease modifying therapy compared to those who were not on disease modifying therapy. Although medications are supplied by the government for free since the hospitals are government supported, the reasons why patients refuse to take these medications remain to be studied. The use of newer medications with less injection frequency and oral medications may lead to improvements in the quality of life.

In our MS patients, quality of life showed an apparent propensity toward a decrease in mean results of EQ-5D and EQ-VAS with age. This observation most likely resulted as a consequence of increased disability throughout the course of the disease and occurrence of more symptoms of MS or other comorbidities that occur with age. Older patients with a progressive and long-term disease appraise their quality of life at a much lower level as compared to younger patients. This indicates that quality of life is mainly influenced by the disability level and disease duration. Our sample was a young one, and future research might capture and compare the same information in an older population.

In conclusion, MS is a disease that is associated with a considerable effect on the patients quality of life. It continues to be challenging to manage both medically and psychosocially. We emphasize the importance of the assessment of quality of life in MS patients. Our results provided insight into a number of associations between patient variables and their quality of life. Healthcare providers should be aware of the low quality of life among patients to improve their quality of life. Clinicians should consider the assessment of quality of life as routine practice along with the other important measures including symptomatic evaluation, laboratory tests, and neuroimaging to provide a holistic care of their MS patients. The results of this study will help in emphasizing the importance of establishing specialized MS clinics, multidisciplinary caring team, and support groups which are currently few in number and limited to big cities.
